# Computer-assisted stereology and automated image analysis for quantification of tumor infiltrating lymphocytes in colon cancer

**DOI:** 10.1186/s13000-017-0653-0

**Published:** 2017-08-29

**Authors:** Ann C. Eriksen, Johnnie B. Andersen, Martin Kristensson, René dePont Christensen, Torben F. Hansen, Sanne Kjær-Frifeldt, Flemming B. Sørensen

**Affiliations:** 1 Danish Colorectal Cancer Center South, Vejle Hospital, Vejle, Denmark; 2 Institute of Regional Health Research, University of Southern Denmark, Odense, Denmark; 3 Department of Clinical Medicine, Stereological Research Laboratory, Aarhus University, Nørrebrogade 44, 10G, 8000 Aarhus C, Denmark; 4 Visiopharm A/S, Agern Allé 24, 2970 Hoersholm, Denmark; 5 Research Unit of General Practice, University of Southern Denmark, J.B. Winsløws Vej 9 A, 1st, 5000 Odense C, Denmark; 6 Department of Pathology, Vejle Hospital, Beriderbakken 4, 7100 Vejle, Denmark; 7 Department of Oncology, Vejle Hospital, Beriderbakken 4, 7100 Vejle, Denmark

**Keywords:** Colon cancer, Heterogeneity, Image analysis, Stereology, Tumor infiltrating lymphocytes

## Abstract

**Background:**

Precise prognostic and predictive variables allowing improved post-operative treatment stratification are missing in patients treated for stage II colon cancer (CC). Investigation of tumor infiltrating lymphocytes (TILs) may be rewarding, but the lack of a standardized analytic technique is a major concern. Manual stereological counting is considered the gold standard, but digital pathology with image analysis is preferred due to time efficiency. The purpose of this study was to compare manual stereological estimates of TILs with automatic counts obtained by image analysis, and at the same time investigate the heterogeneity of TILs.

**Methods:**

From 43 patients treated for stage II CC in 2002 three paraffin embedded, tumor containing tissue blocks were selected one of them representing the deepest invasive tumor front. Serial sections from each of the 129 blocks were immunohistochemically stained for CD3 and CD8, and the slides were scanned.

Stereological estimates of the numerical density and area fraction of TILs were obtained using the computer-assisted newCAST stereology system. For the image analysis approach an app-based algorithm was developed using Visiopharm Integrator System software. For both methods the tumor areas of interest (invasive front and central area) were manually delineated by the observer.

**Results:**

Based on all sections, the Spearman’s correlation coefficients for density estimates varied from 0.9457 to 0.9638 (*p* < 0.0001), whereas the coefficients for area fraction estimates ranged from 0.9400 to 0.9603 (*P* < 0.0001). Regarding heterogeneity, intra-class correlation coefficients (ICC) for CD3+ TILs varied from 0.615 to 0.746 in the central area, and from 0.686 to 0.746 in the invasive area. ICC for CD8+ TILs varied from 0.724 to 0.775 in the central area, and from 0.746 to 0.765 in the invasive area.

**Conclusions:**

Exact objective and time efficient estimates of numerical densities and area fractions of CD3+ and CD8+ TILs in stage II colon cancer can be obtained by image analysis and are highly correlated to the corresponding estimates obtained by the gold standard based on stereology. Since the intra-tumoral heterogeneity was low, this method may be recommended for quantifying TILs in only *one* histological section representing the deepest invasive tumor front.

**Electronic supplementary material:**

The online version of this article (10.1186/s13000-017-0653-0) contains supplementary material, which is available to authorized users.

## Background

Colon cancer (CC) is one of the most common cancers in the Western world [1]. The survival is primarily correlated to the extension of the disease at the time of diagnosis. However, patients diagnosed with the same stage of disease often have markedly different outcomes [2]. This difference in survival rates may be explained by the heterogeneous nature of CC. For a more precise classification of the tumors as well as a better estimation of prognosis, new biomarkers are needed in addition to the current histological grading and the Tumor-Node-Metastasis (TNM) staging system. In this context, tumor infiltrating lymphocytes (TILs) have been analyzed in various settings, and several studies have shown promising results [3]. Numerous inflammatory cells are known to infiltrate malignant tumors, and are considered to be a manifestation of an immunological host reaction against cancer cells, reflecting a tumor-related immune response. The majority of these cells are T-lymphocytes, and in particular cytotoxic T cells.

TILs can be quantified manually or by using automated image analysis. Several scoring methods have been suggested for quantifying TILs, varying from simple classification of presence or absence of peri-tumoral infiltration [4], over the Klintrup-Mäniken score on an hematoxylin and eosin (H&E) stained section [5] to density [6–12] or area-fraction [13] estimation on immunohistochemically stained sections. The lack of a standardized analytic approach is a major concern, and so far none of the proposed techniques for estimating TILs has been incorporated into routine, clinical diagnostic practice.

Manual counting using stereology is considered as the gold standard within quantitative histopathology, although nowadays digital pathology and image analysis are preferred due to time efficiency. An increasing number of studies focus on TILs as a potential prognostic biomarker in CC, and in the recent years digital pathology with the use of image analysis software is emerging for quantifying TILs. In diagnostic pathology the accurate determination of cell counts is of substantial importance, as numerical cut-off values are increasingly used when deciding on the individual patient’s treatment strategy. Several studies have shown that image analysis can detect and determine the density (cells/mm^2^) of TILs in immunohistochemically stained sections, however, only few studies have validated this method [14]. To enable the use of TILs as a clinical biomarker in colorectal cancer, an *Immunoscore* has been proposed [6–8, 15]. The Immunoscore is based on estimates of two different populations of T-lymphocytes counted in two different areas of the tumor defined as the center and invasive front of the tumor, respectively. For prognostic purposes, CD3+ and CD8+ TILs appeared to be most informative, and a multinational investigation of the practical implementation and clinical impact of the Immunoscore is in progress [16]. The combined analysis of tumor center and invasive margin has been suggested to overcome sampling bias caused by heterogeneity. Intra-tumoral heterogeneity is an inherent characteristic of malignant tumors, and it is well known that the micro-environment varies throughout the tumor with effects on growth, proliferation and metastatic potential [17, 18]. This is also the case in CC, which is known to be architecturally, molecularly and biologically heterogeneous [19]. It is well known that tumor heterogeneity may have significant impact on the interpretation of biomarkers [20], but the overall understanding of intra- and inter-tumoral heterogeneity of the micro-environment is limited [21], and to our knowledge the heterogeneity of TILs has only been sparsely investigated in CC. Thus, heterogeneity is of crucial importance in biomarker research, especially in the perspective of clinical application [22].

The aims of this study were to compare manual, stereological estimates of TILs with automatic counts by image analysis. Moreover, we investigated the heterogeneity of TILs in stage II CC in order to decide whether the section of the deepest invasive tumor margin is representative of the whole tumor with regard to TILs.

## Methods

### Patients and tissue

Archival, formalin-fixed, paraffin-embedded (FFPE) CC tissue samples from 44 consecutive patients operated for stage II CC at the Department of Surgery, Vejle Hospital, Denmark in 2002 was retrieved. None of the patients had received preoperative chemo- or radiotherapy. One patient was excluded from the study, since the diagnosis of stage II adenocarcinoma could not be confirmed in the new sections cut. The final study population thus consisted of 43 patients. Mean age was 72.7 years (48–70 years). Six of the tumors were mucinous and the remaining thirty-seven were adenocarcinomas NOS. According to the TNM classification thirty-two of the tumors were classified as T3, nine as T4 and two were unclassified.

The total number of tumor-containing tissue blocks per patient varied from three to 24 (mean = 5.3). Sections of 4 μm thickness were cut from all these blocks (*N* = 229), stained with H&E, and evaluated by first a trainee and then a senior pathologist. Using a ×2.5 or ×5 objective the section representing the deepest invasive front of the tumor was selected. Furthermore, two sections from each patient were selected using a random number table. Thus, each tumor was represented by three sections of the adenocarcinoma.

### Immunohistochemistry

Serial sections were cut from the selected FFPE tumor blocks (*N* = 129) and mounted on FLEX IHC Microscope Slides (K8020, DAKO, Glostrup, Denmark). The pretreatment processes were performed using PT Link (DAKO). Heat-induced epitope retrieval was achieved with Envision Target Retrieval Solution (DAKO) at pH 9 and 97 °C for 20 min.

Staining was performed using a DAKO Autostainer Link 48 (DAKO).

Endogenous peroxidase activity was blocked by Envision FLEX Peroxidase-Blocking Reagent (DAKO). The primary antibodies were mouse monoclonal anti-CD3 (code M7254, DAKO) diluted 1:600, and anti-CD8 (code M7103, DAKO) diluted 1:300. The primary antibodies were diluted with Envision Flex antibody diluent (code S2022 DAKO).

Primary antibodies were incubated for 30 min at room temperature, and for amplification Envision Flex + Mouse(Linker) (DAKO) was used for 20 min. Bound antibodies were detected using Envision FLEX/HRP (DAKO) and visualized by Envision FLEX DAB (DAKO) and chromogene diluted in Envision Flex Substrate Buffer (DAKO). To enhance the immunohistochemical stains, the sections were incubated in 0.5% CuSO_4_ in TBS buffer pH 7.6 for 10 min. Meyer’s hematoxylin (Merck, Damstadt, Germany) was used as counterstain, and finally, the histological slides were coverslipped with Tissue-Tek PERTEX (Histolab Products AB, Göteborg, Sweden).

### Scanning of histological slides and identification of regions of interest

All sections were scanned at 40× magnification using a NanoZoomer XR scanner (Hamamatsu, Japan). The image format was NanoZoomer Digital Pathology Image (*.ndpi) with a resolution of 226 nm/pixel (112,389 dots per inch (DPI), i.e.*,* 4.4 × 4.4 pixels/μm corresponding to a final magnification of ×1.558).

**Fig. 1:**
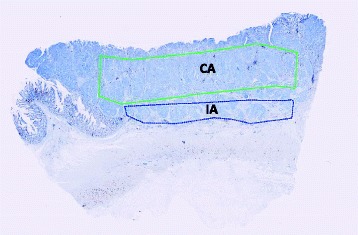
Region of interest. For each tissue section the region of interest (ROI) was manually marked. Green line demarcates central tumor area (CA) and blue line the invasive area (IA), including the invasive front of the adenocarcinoma

**Fig. 2:**
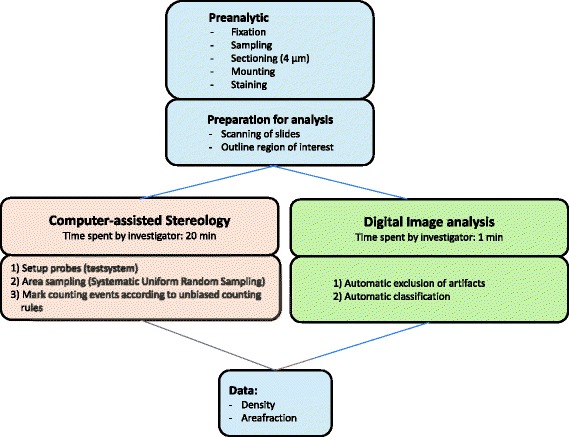
Workflow for stereology and image analysis. The regions of interest were outlined manually and the exact same areas were used for both technical approaches. The stereological analysis was performed, using a computer assisted software. The field of views were selected by the software by systematic random sampling, while the counting was carried out manually by the observer. The image analysis was performed automatic using, an image analysis algoritm

The quantification was performed using Visiopharm Integrator System software (VIS; Visiopharm A/S, Hoersholm, Denmark) and hardware Lenovo ThinkPAD W541 core i7 16GB (Morrisville, North Carolina, USA). Regions of Interest (ROIs) were manually delineated by the observer in the software as a central area (CA) and an invasive area (IA) (Fig. 1). These outlined areas were used for both the stereological analysis and the digital analysis. The entire tissue handling and processing is summarized in Fig. 2. The CA was outlined as the central part of the adenocarcinoma, not including areas with budding or irregular tumor islands. The lumen and the luminal surface of the tumor and areas with adenoma were also avoided. The IA was outlined including the outermost 1/5 of the invasive front of the adenocarcinoma, including the deepest invasive front of the tumor, areas with tumor budding or irregular tumor islands. For very small tumors we outlined the 1/10 outermost part of the invasive front as the IA. We only outlined a small rim of the stoma in tumors with pushing borders, while in tumors with infiltrative borders a greater part of stoma in between the irregular tumor islands and budding cells, was included. Areas with necrosis, mucin, artificial clefts or tissue folds were avoided. Also, follicular lymphoid aggregates with germinal centers were excluded from evaluation.

### Stereological analysis

**Fig. 3:**
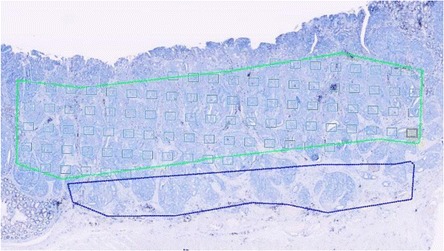
Systematic, uniform random sampling of fields of vision using newCAST software. The yellow frame represents the current field of vision (FOV)

**Fig. 4:**
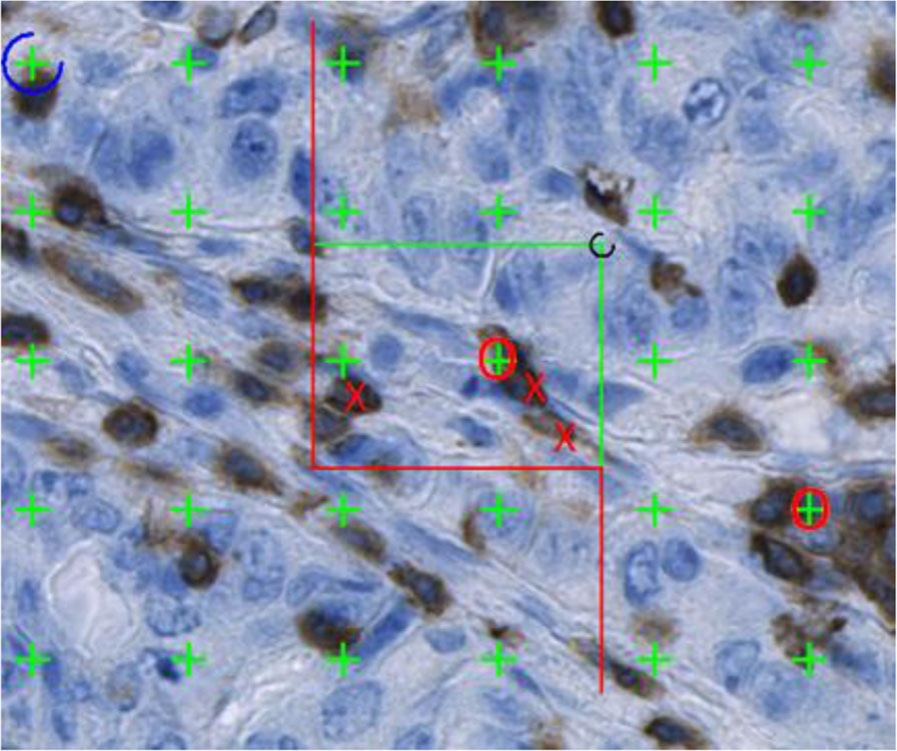
Field of vision in a CD3-stained section magnified ×40. The density estimation was performed using the 2D unbiased counting frame with left and bottom edges, and their extensions, serving as exclusion lines (red), and with the upper and right edges of the frame as inclusion lines (green). Cell profiles were counted when completely inside the counting frame or partly inside the frame, provided that they did not touch the exclusion lines or their extensions. Thus, three cell profiles were counted (red crosses). The area fraction estimation was performed using the point grid. Points hitting CD3 positive cells = 2 (red ring) and points hitting tumor = 30, giving an area fraction of 0.07 in this field of vision

The stereological analysis was performed, using the computer assisted stereology software newCAST (Visiopharm A/S). The computer selected the fields of view (FOV) by systematic, random sampling within the ROI (Fig. 3). The sampling fraction varied according to the size of the ROI and the lymphocytic density. We aimed to count a minimum of 200 immunohistochemically CD3+ and CD8+ positive lymphocytes in each tumor, and a pilot study showed that according to a low, medium or high lymphocytic density, this could roughly be obtained by 100, 40, and 25 FOVs, respectively. The cells were counted using an integrated test-system consisting of a 2D unbiased counting frame and a point grid [23]. The screen magnification used for counting was ×40. Cell profiles to be counted were defined as lymphocytes with discernible nucleus and immunohistochemically positive membranous/cytoplasmic staining for CD3 or CD8, respectively. Cell profiles, where no nucleus could be distinguished, were not counted. Profiles were counted according to the 2D unbiased counting rule [23], in that the counting frame areas were adapted to 1.575 μm^2^ and 2.925 μm^2^ for CD3 and CD8 TILs estimation, respectively, taking into account the different densities of the two lymphocytic populations (Fig. 4).

The corner point in the counting frame was counted (P), when hitting vital tumor tissue, but not counted when hitting necrosis, mucin, artificial clefts or tissue folds. The number of profiles *per* area (Q_A_ = *numerical density)* was calculated as the number of positive lymphocytic profiles divided by the total, investigated counting frame area [24]:

$$ {Q}_A\left( prof/ sect\right)=\frac{\sum Q(prof)}{\sum P\bullet counting frame area}=\frac{\sum \# of positive lymphocytic profiles}{total sampling area} $$

The *area fraction* was estimated using the points (crosses) of the integrated grid. A point was counted as positive every time the upper right corner of the cross was covered by a viable CD3+ or CD8+ lymphocyte, using the same definitions of membranous/cytoplasmic and nuclear staining as mentioned above. A point was counted as negative every time the upper right corner of the cross covered immuno-negative cells or stroma. Points falling into areas of muscle tissue, necrosis, mucin, vessels, artificial clefts, or tissue folds were not counted. Each point on the grid is associated with an area, and the total area is estimated by multiplying the total number of points collected across the entire ROI by the area associated with a point. For the CD3+ and CD8+ area estimation, the area per point was 559.69 μm^2^. The area fraction was calculated dimensionless as the sum of points hitting the CD3+ or CD8+ lymphocytes divided by the sum of the points hitting the vital tumor tissue within the ROI [24]:

$$ Area fraction=\frac{\sum {P}_{CD3+}{/}_{CD8+ lymphocytes}}{\sum {P}_{tumor}} $$

### Image analysis

Using Visiopharm Quantitative Digital Pathology software (Visiopharm A/S), an App-based image analysis algorithm was developed specifically for counting of CD3+ and CD8+ lymphocytes. A training set was used to configure the algorithm and identify immuno-positive lymphocytes. The analysis of the training set was performed blinded and the estimated number of positive lymphocytes in each sample was subsequently compared to the manual, stereological evaluation performed by the pathologist. The algorithm included two image processing steps:

1. Automatic exclusion of artifacts, including tissue folds and clefts, mucin, fatty tissue and necrosis to avoid contributions from regions of no interest.
2. Automatic classification of CD3+ or CD8+ lymphocytes using a Bayesian classifier.

The algorithm sampled and analyzed the whole ROI. The first image processing step involved a segmentation of the outlined area using a linear Bayesian pixel classifier on a mean filtered intensity representation of the image. This was performed at ×1 magnification, digitally created by the software. By limiting the magnification the amount of data are decreased and thus the processing speed is increased. A priori knowledge of the tissue was used to post-process the identified artefact objects.

**Fig. 5:**
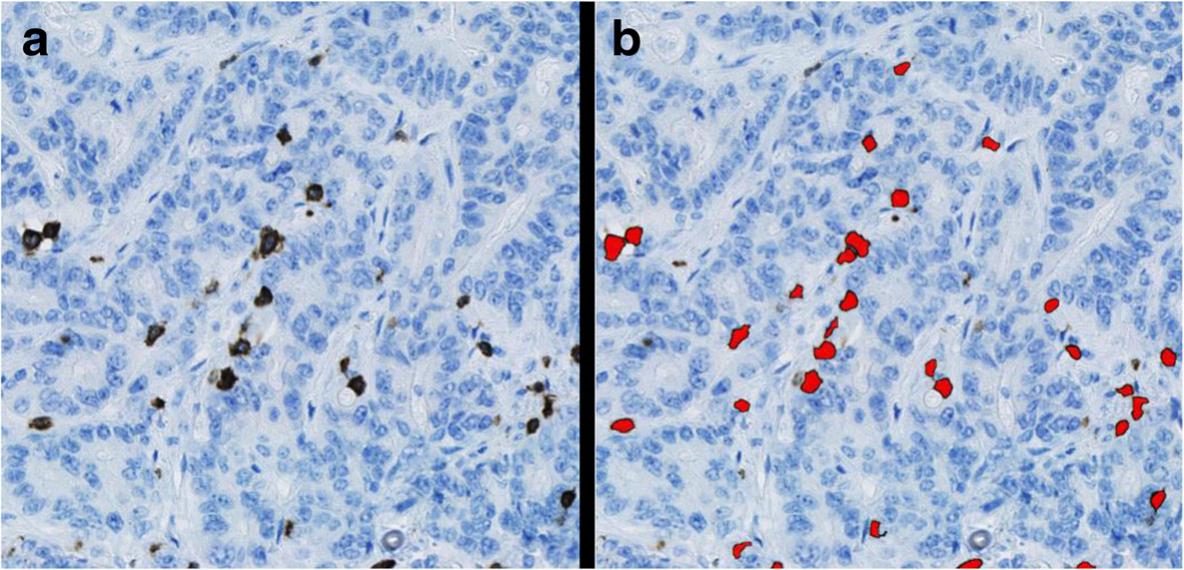
Example of the processing of the image analysis. **a**) Part of a CD3 stained section. **b**) CD3+ lymphocytes labelled with the red color are counted by the software

In the second step, a high-resolution analysis of the identified tissue areas was performed using a Bayesian classifier trained on pre-processing steps that highlight the red and blue chromaticity, and local circular objects were enhanced using Visiopharms Polynomial Blob filter (Visiopharm A/S). This detects CD3+ or CD8+ lymphocytes and performs an automatic count of the positive objects (Fig. 5). Object based post-processing was applied to remove negative objects, weakly stained objects, and separate objects using a built-in watershed function.

### Statistical analysis

Data were summarized and inspected using standard statistical methods. By visual inspection of QQ-plots it was found that the log-transformed data were normally distributed. Inter- and intra-tumoral variability was assessed by calculation of intra-class correlation coefficients (ICC) [25] using a mixed-effects model defining ICC as a post-estimation command. In this setting ICC is a ratio of variance and considered as the percentage of the total variance accounted for by the differences among the tumors examined. The ICC will be high (ICC → 1), if the majority of the estimator variation is attributable to inter-tumoral variation (=biological variation among the patients). In case the majority of variation is caused by intra-tumoral variation (i.e.*,* heterogeneity), the ICC will, however, be low (ICC → 0).

The correlations between the manually obtained stereological estimates and the image analysis counts were evaluated for the individual tissue sections and for mean values of individual tumors, based on all three tumor sections, using the Spearman correlation coefficient.

*P*-values less than 0.05 were considered significant and all tests were 2-sided. The statistical analysis was performed using the software STATA version 14.0 (StataCorp, Texas, USA).

## Results

The outlined ROIs varied from 3.7 to 87.4 mm^2^ (mean 20.7 mm^2^) for the CA, and from 1.7 to 43.4 mm^2^ (mean 12.2 mm^2^) for the IA.

### Correlation between image analysis and stereology

#### Density

The CD3+ numerical T-cell densities in CA varied from 50 to 1786 cells/mm^2^, when counted stereologically, while image analysis ranged from 53 to 1680 cells/mm^2^. In the IA the CD3+ numerical T-cell densities varied from 60 to 2302 cells/mm^2^ by stereology and from 57 to 1927 cells/mm^2^ by image analysis. In the CA, the CD8+ numerical T-cell densities varied from 8 to 2043 cells/mm^2^ by stereology, while image analysis ranged from 18 to 2195 cells/mm^2^. In the IA the CD8+ numerical T-cell densities varied from 17 to 1852 cells/mm^2^ and from 24 to 1852 cells/mm^2^ by stereology and image analysis, respectively (Table 1).

**Table 1:** Numerical density estimates

	Mean	Min	Max
CD3-Central Image analysis	557	53	1680
CD3-Central Stereology	533	50	1786
CD3-Invasive Image analysis	373	57	1927
CD3-Invasive Stereology	402	60	2302
CD8-Central Image analysis	375	18	2195
CD8-Central Stereology	285	8	2043
CD8-Invasive Image analysis	398	24	1695
CD8- Invasive Stereology	360	17	1852

Numerical density estimates of CD3+ and CD8+ tumor infiltrating lymphocytes *per* mm^2^ in central and invasive tumor areas, respectively, as obtained by either stereology or image analysis (*n* = 129)

**Fig. 6:**
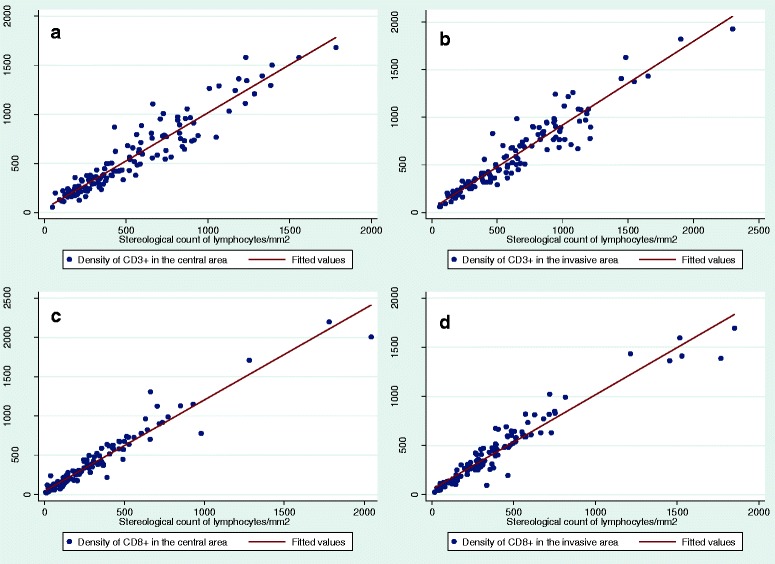
Correlation between cell counts as obtained by stereology and image analysis. **a**) Correlation for CD3+ tumor infiltrating lymphocytes (TILs) numerical density in the central area of the tumor. **b**) Correlation for CD3+ TILs numerical density in the invasive tumor front. **c**) Correlation for CD8+ TILs numerical density in the central area of the tumor. **d**) Correlation for CD8+ TILs numerical density in the invasive tumor front

The lymphocytic density counts obtained by image analysis were highly correlated with the stereologically based estimates from the same images (Fig. 6). The correlation was analyzed for all sections (*N* = 129) and for each of the three individual sections from each tumor, i.e.*,* the section with the deepest tumor penetration and the two randomly selected sections, and all correlations were optimal. The Spearman correlation coefficients for density counts varied from 0.9496 to 0.9638, when analyzed for all sections. When only considering the tissue section with the deepest tumor invasion, the correlation coefficient ranged from 0.9609 to 0.9784, and for the randomly chosen tissue sections the correlation coefficient ranged from 0.9328 to 0.9745 for section A and from 0.9080 to 0.9644 for section B (Table 2).

**Table 2:** Correlation between central and invasive area for density estimates

	Spearman correlation All sections (*n* = 129)	Spearman correlation Deepest section (*n* = 43)	Spearman correlation Random section A (*n* = 43)	Spearman correlation Random section B (*n* = 43)
CD3-Central	0.9457	0.9623	0.9328	0.9080
CD8-Central	0.9638	0.9609	0.9745	0.9431
CD3-Invasive	0.9496	0.9678	0.9420	0.9644
CD8-Invasive	0.9552	0.9782	0.9367	0.9381

Correlation between numerical densities of CD3+ and CD8+ tumor infiltrating lymphocytes *per* mm^2^ in central and invasive tumor areas, respectively, estimated by either stereology or image analysis for all tumor sections and for individual tumor sections. Spearman’s correlation, *p* < 0.0001

#### Area fraction

In the CA, the estimates of area fraction of CD3+ T-cells varied from 0.15 to 6.67% by stereology, while image analysis ranged from 0.28 to 9.44%. In the IA the CD3+ T-cell area fractions varied from 0.16 to 6.77% by stereology and from 0.27 to 9.43%, when estimated by image analysis. Estimates of the area fractions of CD8+ T-cells in the CA varied from 0.03 to 13.59% by stereology, while ranging from 0.11 to 15.01% by image analysis. In the IA the CD8+ T-cell area fractions varied from 0.08 to 13.33%, when estimated by stereology, and from 0.17 to 14.07% by image analysis (Table 3).

**Table 3:** Area fractions

	Mean	Min	Max
CD3- Central Image analysis	2.96	0.28	9.44
CD3-Central Stereology	1.90	0.15	6.67
CD3-Invasive Image analysis	3.11	0.27	9.43
CD3-Invasive Stereology	2.12	0.16	6.77
CD8-Central Image analysis	2.62	0.11	15.01
CD8-Central Stereology	1.83	0.03	13.59
CD8-Invasive Image analysis	2.84	0.17	14.07
CD8-Invasive Stereology	2.32	0.08	13.33

Area fractions (%) of CD3+ and CD8+ tumor infiltrating lymphocytes in central and invasive tumor areas, respectively, as obtained by either stereology or image analysis (*n* = 129)

**Fig. 7:**
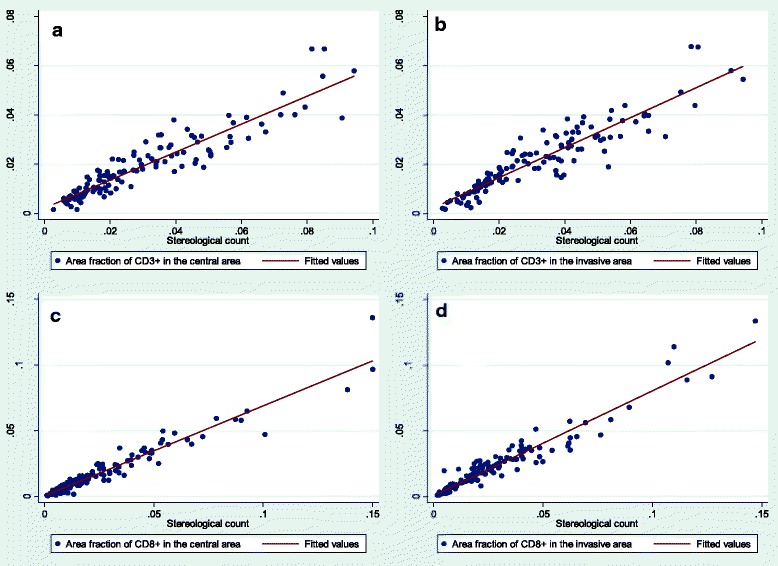
Correlation between area fractions as estimated by stereology and image analysis. **a**) Correlation for CD3+ tumor infiltrating lymphocytes (TILs) area fraction in the central tumor area. **b**) Correlation for CD3+ TILs area fraction in the invasive tumor front. **c**) Correlation for CD8+ TILs area fraction in the central tumor area. **d**) Correlation for CD8+ TILs area fraction in the invasive tumor front

Similar to the results of the numerical densities, the area fraction estimation by image analysis had a high correlation with the stereological estimation from the same images (Fig. 7). The Spearman correlation coefficients for area fractions varied from 0.9400 to 0.9603, when analyzed for all sections. Considering only the section representing the deepest invasive front, the correlation coefficients ranged from 0.9406 to 0.9665, and for the randomly chosen sections from 0.9080 to 0.9671 for section A and from 0.9244 to 0.9710 for section B (Table 4).

**Table 4:** Correlation between central and invasive area for estimates of area fraction

	Spearman correlation All sections (*n* = 129)	Spearman correlation Deepest section (*n* = 43)	Spearman correlation Random section A (*n* = 43)	Spearman correlation Random section B (*n* = 43)
CD3-Central	0.9404	0.9434	0.9322	0.9244
CD8-Central	0.9603	0.9499	0.9671	0.9269
CD3-Invasive	0.9400	0.9406	0.9394	0.9710
CD8-Invasive	0.9497	0.9665	0.9080	0.9557

Correlation between area fractions of CD3+ and CD8+ tumor infiltrating lymphocytes in central and invasive tumor areas, respectively, estimated by either stereology or image analysis for all tumor sections and for individual tumor sections

Spearman’s correlation, *p* < 0.0001

In general, the correlation coefficients are close to 1, implying a very high correlation between estimates of TILs densities and area fractions obtained by either the manual, stereological technique or image analysis. For both numerical density and area fraction the correlation coefficients increased, when only considering the section representing the deepest invasive front, except for the count of CD8+ TILs in CA.

#### Intra-observer / intra-technical reliability

Estimates of the numerical density and the area fractions, as obtained separately by stereology and image analysis, respectively, showed a high reproducibility (Table 5). The intra-observer reproducibility test of the stereological method showed correlation coefficients varying from 0.9575 to 0.9676, and for the “intra-technical reproducibility test” of the image analysis the correlation coefficients varied from 0.9890 to 0.9926.

**Table 5:** Correlation between numerical density and area fraction

	Spearman correlation All sections (*n* = 129)	Spearman correlation Deepest section (*n* = 43)	Spearman correlation Random section A (*n* = 43)	Spearman correlation Random section B (*n* = 43)
CD3-Central Stereology	0.9575	0.9503	0.9688	0.9448
CD3-Central Image analysis	0.9926	0.9872	0.9878	0.9899
CD8-Central Stereology	0.9796	0.9838	0.9578	0.9872
CD8-Central Image analysis	0.9932	0.9869	0.9941	0.9917
CD3-Invasive Stereology	0.9519	0.9457	0.9443	0.9438
CD3-Invasive Image analysis	0.9890	0.9905	0.9787	0.9962
CD8-Invasive Stereology	0.9676	0.9665	0.9713	0.9565
CD8-Invasive Image analysis	0.9907	0.9917	0.9912	0.9878

Correlation between numerical densities and area fraction obtained by either stereology or image analysis for all tumor sections and for individual sections. Spearman’s correlation, *p* < 0.0001

#### Evaluation of efficiency

The time spent on outlining ROIs was approximately 3 min, which applies to both methods. Subsequently, an experienced observer spent an average of 20 min *per* tissue section on the stereological method for the estimation in both CA and IA. This included set-up of probes, systematic random sampling in the ROIs, and counting of cell densities and area fractions. In contrast, the investigator time spent on the digital image analysis was 3 min for outlining a ROI plus less than 1 min and for queuing up the slide for further analysis. The analysis by the computer required 10–15 min per slide analyzing 100% of the region of interest, with no need for hands on by the pathologist. Thus, image analysis of the tissue sections could be running overnight with no observer interaction needed.

### Heterogeneity

**Fig. 8:**
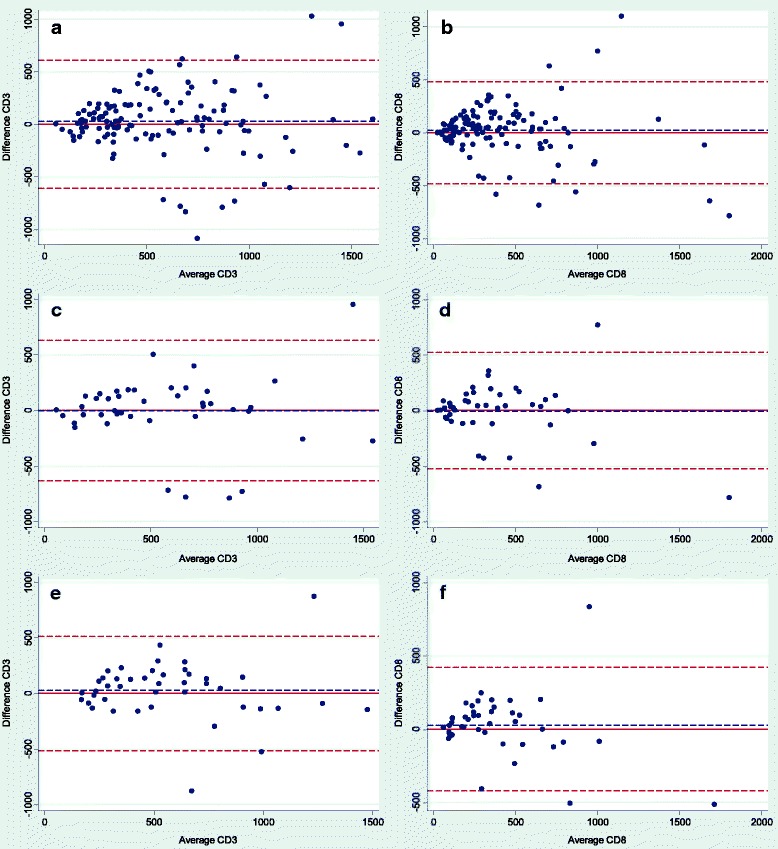
Bland Altman plots showing the differences in densities of CD3+ and CD8+ tumor infiltrating lymphocytes for the central and invasive area measured by image analysis. The horizontal red line corresponds to zero difference, the blue dashed line shows mean and the dashed red lines show ±1.96 standard deviation. **a** and **b**) Differences for densities of CD3+ and CD8+ tumor infiltrating lymphocytes (TILs) for all sections (*n* = 129). **c** and **d**) Differences for densities of CD3+ and CD8+ TILs for “the deepest invasive sections” (*n* = 43). **e** and **f**) Differences for densities of CD3+ and CD8+ TILs per tumor, where density is calculated as an average of the densities obtained from each of the three sections from each tumor (*n* = 43)

Overall we found a tendency of higher densities of both CD3+ and CD8+ TILs in the invasive area compared to the central area. This was also found when only considering the deepest invasive section (Fig. 8).

The ICCs were calculated for the CA and IA of the adenocarcinomas for estimates obtained by both the manual, stereological technique and the image analysis. According to the numerical densities for CD3+ TILs, the ICC varied from a minimum of 0.665 in the CA by stereology to a maximum of 0.712 found in the CA by image analysis (Table 6). For CD8+ TILs the ICC varied from 0.765 in the IA by both the stereological method and the image analysis to 0.775 in the CA of the adenocarcinoma by stereology.

**Table 6:** Intra-class correlation coefficient for numerical density estimates

	ICC (CD3)	ICC (CD8)
Image analysis Central	0.712 (0.613–0.810)	0.749 (0.661–0.838)
Stereology Central	0.665 (0.555–0.776)	0.775 (0.693–0.856)
Image analysis Invasive	0.686 (0.581–0.792)	0.765 (0.681–0.849)
Stereology Invasive	0.707 (0.607–0.807)	0.765 (0.682–0.849)

Intra-class correlation coefficients (ICC) and 95% confidence intervals (CI) for estimates of numerical density of CD3+ and CD8+ tumor infiltrating lymphocytes in central and invasive tumor areas, respectively, as obtained by stereology or image analysis

Similar results were found calculating the ICC based on the estimates of area fractions (Table 7). For these estimates the ICC for CD3+ TILs varied from a minimum of 0.615 in the CA by stereology to 0.746 found in the IA also by stereological counts. For estimates of the CD8+ TILs area fraction, the ICC varied from 0.724 to 0.763.

**Table 7:** Intra-class correlation coefficient for estimates of area fraction

	ICC (CD3)	ICC (CD8)
Image analysis Central	0.704 (0.603–0.804)	0.746 (0.657–0.836)
Stereology Central	0.615 (0.493–0.737)	0.724 (0.628–0.819)
Image analysis Invasive	0.702 (0.601–0.804)	0.763 (0.678–0.847)
Stereology Invasive	0.746 (0.657–0.836)	0.746 (0.657–0.835)

Intra-class correlation coefficients (ICC) and 95% confidence intervals (CI) for estimates of area fractions of CD3+ and CD8+ tumor infiltrating lymphocytes in central and invasive tumor areas, respectively, as obtained by either stereology or image analysis

According to the calculated values of ICC, the majority of the variation is due to biological differences among the tumors/patients. Taken together, the ICCs ranged in the interval from 0.615 to 0.775 for both numerical densities and area fractions. This means that 61.5% to 77.5% of the total estimator variation can be attributed to differences among tumors, whereas 38.5% to 32.5% of the total variation is caused by variation within the single tumor (i.e. heterogeneity and measurement noise). Thus, the intra-tumoral variation is considerably lower than the inter-tumoral variation, when considering the central and invasive area separately. We also calculated ICCs including all six sections from each tumor, which revealed ICCs of 0.578 (95%CI: 0.445–0.710) for CD3 and 0.603 (95%CI: 0.478–0.729) for CD8 respectively. Thus the intratumoral heterogeneity was increased, indicating variation between the invasive and central areas.

Staining intensity histograms and ascending standard deviation plot (Additional file 1) show similar intensities in the different compartments for CD3 and CD8, respectively; however there is a clear difference between intensities for CD3 and CD8.

## Discussion

In this study we quantified CD3+ and CD8+ TILs using stereology and image analysis in adenocarcinomas of the colon, and we investigated the intra-tumoral heterogeneity of TILs. We found an excellent correlation between estimates obtained by the manual, stereological technique and the image analysis. Additionally, we found that the intra-tumoral variation is considerably lower than the biological variation among tumors.

### Correlation between image analysis and stereology

The slight variation between the stereology count and the image analysis was mainly a result of either weak immunohistochemical staining of the T-cells, where the image analysis may miss lymphocytes, or non-specific background staining (i.e. noise), which may be misinterpreted by the image analysis software as lymphocytes. Thus, the image analysis does not consequently over- or underestimate TILs but is rather dependent on the staining quality of the individual section. In some sections we saw an almost perfect correlation between image analysis and stereology in the CA, however, in the IA the correlation was weaker due to background noise in this particular compartment. The image analysis algorithm was designed using images with different staining intensities, and it is not possible to completely avoid misinterpretation of the stained objects. Inclusion of weakly stained lymphocytes may lead to a higher sensitivity for background noise and vice versa. Basically, the aim of an image analysis algorithm is to remove disturbing features and to enhance the structures of interest, and this can be achieved in many different ways. In the present setting background noise was removed using a mean filter, and for enhancing the lymphocytes we used a polynomial blob filter followed by classification by a Bayesian classifier.

To avoid misclassification the quality of the immunostaining is crucial. Our definition of a positive cell profile for the stereological estimation was a clear cut immunostaining of the cytoplasm/membrane for CD3 or CD8, and a discernible nucleus. To obtain an optimal immunostaining, we only used validated antibodies recommended by NordiQC [26]. However, we are aware that we did not have any influence on any pre-analytical confounders, such as tissue fixation and processing, which may have considerable impact on the quality of the immunohistochemical staining. This is a weakness associated with all studies using a retrospective design, and a prospective study would be warranted. Section thickness may vary a bit, even when using state of the art microtomes, and this variation in thickness is an argument for not including intensity in the evaluation process. In some cases the image analysis software failed to complete delete areas of mucin or necrosis, but most often the areas were excluded correctly. Furthermore, we investigated the impact of the mucinous component in the six mucinous adenocarcinomas included in our series of tumors by performing a sensitivity analysis excluding the sections of mucinous CC (*n* = 18). This resulted in almost unchanged correlation coefficients varying from 0.9497 to 0.9633 (*p* < 0.0001; data not shown). The image analysis algorithm did not handle exclusion of muscle tissue and vessels, which may partly explain the discrepancy with the stereological estimation. However, this accounts for very small areas, since this only represents a minor part of the tumors.

Our results are in agreement with a study comparing image analysis with manual, stereological estimates of TILs in early stage cervical cancer [27], but to our knowledge the present study is the first to comparatively investigate these techniques in CC. Carus et al. [27] found a significant, but lower correlation between the two techniques in obtaining estimates of CD8+ lymphocytes. This might be explained by the fact that their image analysis yielded an area fraction, whereas their stereological approach was based on numerical density. Thus, the two estimates, obtained in their study, were not directly comparable.

Väyrynen et al. [14] performed manual, semi-quantitative estimation of CD3+ lymphocytes on captured images in 34 randomly selected cases of colorectal cancer and found numerical densities varying from 1.8 to 2243 cells/mm^2^ (median 471 cells/mm^2^). They compared with computer-assisted image analysis and found correlation coefficients very similar to our results, varying from 0.960 to 0.987. Their manual counts were not based on stereology, and moreover, they were performed in a limited number of fields of vision without a clearly stated sampling approach. The counts were, however, performed by four different observers. We only had one observer, but using strict stereological counting rules our results were reproducible by both techniques. We investigated the correlation between the numerical density estimates and the area fractions, as obtained separately by the stereological technique or image analysis, and found excellent correlation coefficients for these “intra-technical reproducibility tests”.

Stereology is considered the gold standard for histopathological quantification; however, we did not perform unbiased stereology. Unbiased estimation of cell quantity would require a 3D probe, e.g. using a disector, where the third dimension is taken into account [28]. Such an approach would be very time consuming, and since our aim was to compare the stereological estimates with counts obtained by image analysis, we counted TILs only in 2D, i.e. profile counting. A lymphocyte has an average diameter of 8–10 μm, and to avoid missing cells we used 4 μm thick tissue sections. Thicker sections may both in the stereological technique and image analysis give difficulties in counting overlapping cells, especially in tumors with high numbers of infiltrating TILs. Halama et al. [29] focused their study on the count of lymphocytes in conglomerates and they used 2 μm thick sections to minimize the possible overlap between cells. They found a high inter-observer variation for manual counts, especially for tumors with high number of TILs. Our concern with 2 μm sections would be problems with a clear nuclear definition. Due to the issue of overlapping cells we also estimated TILs by area fractions. When comparing the stereological estimates of area fraction with those obtained by image analysis, we found a slightly lower correlation compared to the numerical density estimates, but overall the correlation was nearby optimal.

Image analysis provides objective quantitative measurements and is known to have a high reproducibility (i.e. precision), which makes it highly valuable in terms of standardization. According to the algorithm design, the same result can be produced again on the same image. However, accuracy of the measurement, which is defined as the closeness of agreement between a measured value and the true value, is also important to take into consideration. Image analysis can provide precise and reproducible results, which may, however, be biased. Generally, image analysis is validated by semi-quantitative, manual evaluations that may be associated with considerable inter- and intra-observer variability, even among trained pathologists. Such validations could therefore lead to a systematic skewness or bias of the results, and this is the main reason why we choose to compare our image analysis algorithm with stereology, which has both a high precision and accuracy. This is especially important, when considering cut-off levels for the triage of therapy. Moreover, most papers [6–8, 10–13] do not report validation, and thus the obtained estimates of TILs might be biased and difficult to compare to other studies. Stereology and image analysis both produce numerical data, which have the advantage of easy comparability with results obtained by the use of another software. Use of semi-quantitative approaches might inhibit or make comparisons with other studies difficult.

The stereological approach has an inherent subjectivity, in that the investigator needs to decide what to count or not to count, but the use of well-defined counting rules and immunohistochemical stains reduces this subjectivity to a minimum. Image analysis is non-subjective. Both methods are considered robust and reproducible. The only observer bias might be associated with the subjective outlining of the region of interest, however our clear definition minimizes this source of bias.

The ongoing *Immunoscore* project has the aim of standardizing the procedure for quantifying TILs, and the scientific research group advocating the Immunoscore also recommends automatic quantification of TILs [15, 16]. Similar to the Immunoscore project, we analyze CD3 and CD8 positive TILs in two different areas of the tumor. However, a difference between the studies might be the definition of the sampling areas, and this is highlighted as the time consuming part for the pathologist [30]. Moreover, we have not been able to find an exact description of the sampling approach (i.e., area selected) used in the Immunoscore project. Although stereological methods have evolved over the years to be more efficient, they are still laborious and time consuming. In our study the stereological counts required an average of 20 min per section. In contrast, the image analysis method required less than one minute by the investigator. The subsequent automatic image analysis took 10–15 min per slide (analyzing 100% of the region of interest), but the analysis could be performed at any time, day or night, without investigator assistance. Thus, automated digital image analysis requires less human resources than the manual stereological approach, which is in agreement with Ong et al.*,* who found the use of computer-assisted, pathological immunohistochemical scoring time-saving compared to conventional visual semi-quantitative scoring [31].

### Heterogeneity

We investigated the heterogeneity of TILs in both the CA and the IA of the adenocarcinomas, well aware that this only represents a minor part of the whole tumor. Dealing with a retrospective design, it was not possible to overcome sampling bias, since the investigated tissue had already been sampled and prepared for diagnostic purposes. Many studies on heterogeneity have used tissue micro-arrays (TMAs), and depending on the core diameter, the analyzed tumor area varies from 0.28 mm^2^ [8] to 3.14 mm^2^ [9]. We used three whole sections from each tumor and analyzed a considerably larger tumor bulk than TMA-based studies.

Intra-tumoral heterogeneity may lead to sampling bias, and it is important to take this into account in estimating TILs, especially with the perspective of clinical, diagnostic implementation. Some studies evaluate TILs by hot spot sampling [6, 7, 12], while others evaluate in randomly selected tumor areas [10]. To overcome sampling bias due to intra-tumoral heterogeneity, several studies investigating TILs have focused on different tumor compartments, e.g. the Immunoscore, which combines analysis of TILs in the tumor center and at the invasive tumor margin [5–7]. This may overcome the heterogeneity between the tumor center and the invasive front but does not take into account the heterogeneity found solely in the central and/or invasive front of the tumor. Galon et al. [8] measured CD3+ and CD8+ TILs in duplicates of spots representative of the tumor center and invasive tumor front. They documented a high level of homogeneity in each tumor region, but it was not reported, whether the sampling was based on hot spots or randomly selected FOV. Also, Nosho et al. [11] investigated heterogeneity by taking two-four TMA cores from each tumor, but it did not appear from which part of the tumor these cores were taken. Despite the investigation of several cores form each patient, none of these studies present data on intra-tumoral heterogeneity, and the reported results on the prognostic impact of TILs are inconsistent [8, 11].

Overall, the heterogeneity of TILs in CC has only been sparsely investigated. The study most similar to ours was performed by Laghi et al. [13], who measured CD3+ T-cells in three random and non-contiguous microscopic areas representing the deep front of tumor invasion. Their investigation was restricted to one tissue section from each tumor and TILs were quantified solely as area fractions. However, they found homogenous results in 66% of the tumors, which is in agreement with our results. In the invasive tumor front we found ICC for CD3+, quantified as area fractions, to be 0.702 by the image analysis method and 0.746 by the stereological technique.

In summary, we demonstrated that biological inter-tumoral variation contributes to the overall variation of TILs estimates to a much higher degree than intra-tumoral heterogeneity. It may therefore be rational only to quantify TILs in one section. For the purpose of reproducibility and comparability we recommend investigating the section representing the deepest invasive tumor front.

## Conclusion

We found an excellent correlation between the manual, stereological technique and the app-based image analysis, both in the CA and in the IA, for estimation of CD3+ and CD8+ lymphocytic densities in histological sections from CC. The computer assisted, app-based image analysis is a fast and efficient method of quantifying TILs in CC and has potential of routine application. The image analysis algorithm may be sensitive to variation in staining protocols, calling for inter-laboratory reproducibility studies. Being robust as to heterogeneity, we recommend quantifying TILs in CC using one tissue section representing the deepest invasive area of the adenocarcinoma.

## Additional file

Additional file 1: Figure S1. A. Histogram of intensity levels for all positive detected nuclei in both the central and invasive area. B. Ascendingly plotted standard deviations per tumor (3 sections). (DOCX 73 kb)

## Abbreviations

CA: Central area
CC: Colon cancer
FOV: Field of view
H&E: Hematoxylin and eosin
IA: Invasive area
ICC: Intra-class correlation coefficient
ROI: Regions of interest
TILs: Tumor infiltrating lymphocytes
