# Supplementary material for: Computer-assisted stereology and automated image analysis for quantification of tumor infiltrating lymphocytes in colon cancer
Source: Diagn Pathol. 2017 Aug 29;12:65. doi: 10.1186/s13000-017-0653-0 (PMC5575870; doi:10.1186/s13000-017-0653-0)
Supplement: Additional file 1: Figure S1. — A. Histogram of intensity levels for all positive detected nuclei in both the central and invasive area. B. Ascendingly plotted standard deviations per tumor (3 sections). (DOCX 73 kb) [file 13000_2017_653_MOESM1_ESM.docx]

**Figure S1**

**A.** Histogram of intensity levels for all positive detected nuclei in both the central and invasive area.

**B.** Ascendingly plotted standard deviations per tumor (3 sections).


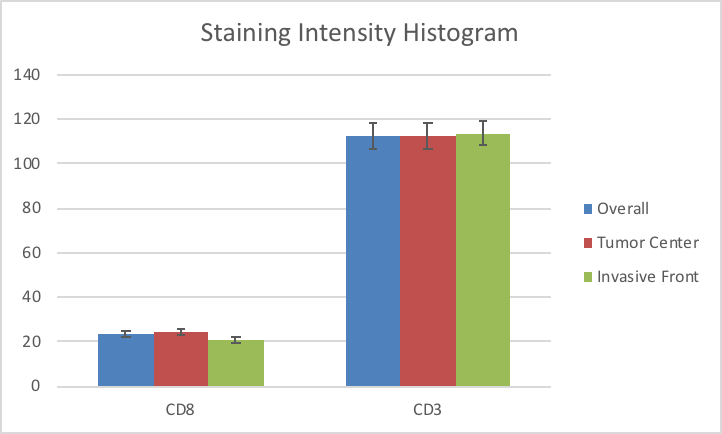


A


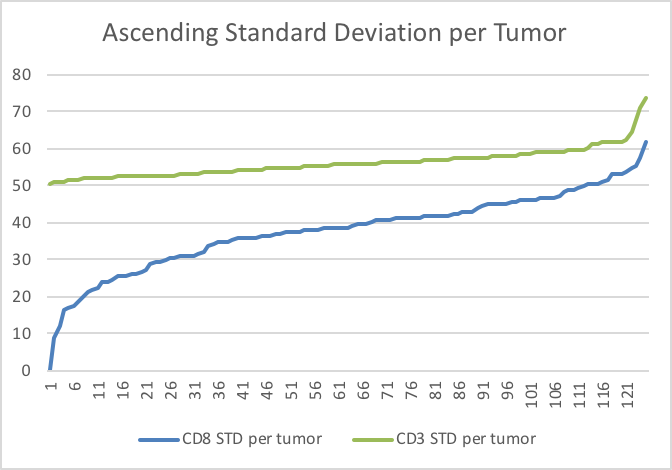


B
